# Beyond a cure for TB: confronting the hidden burden of post-TB lung disease

**DOI:** 10.5588/ijtldopen.25.0222

**Published:** 2025-05-12

**Authors:** Y. Kherabi, D.R. Silva, R. Centis, L. D’Ambrosio, G.B. Migliori

**Affiliations:** ^1^ Infectious and Tropical Diseases Department, Bichat-Claude Bernard Hospital, Assistance Publique Hôpitaux de Paris, Université Paris Cité, Paris, France;; ^2^ Université Paris Cité, Inserm, IAME, Paris, France;; ^3^ Faculdade de Medicina, Universidade Federal do Rio Grande do Sul (UFRGS), Porto Alegre, Brasil;; ^4^ Servizio di Epidemiologia Clinica delle Malattie Respiratorie, Istituti Clinici Scientifici Maugeri IRCCS, Tradate, Italy;; ^5^ Public Health Consulting Group, Lugano, Switzerland.

## Abstract

Although much attention is given to diagnosis and treatment for TB, recent evidence highlights the suffering that occurs after patients are ‘cured’. This issue of *IJTLD Open* includes two studies that improve our understanding of the long-term impact of TB. First, a study in Kenya, Uganda, Zambia and Zimbabwe evaluated the feasibility of assessing and referring TB patients for comorbidities, risk factors and disabilities within national TB programmes. Second, a study of patients who successfully completed TB treatment in China sheds light on the long-term sequelae of TB. The combined findings reinforce the need to integrate care strategies into TB programmes to address these health challenges.

TB is the leading cause of death from an infectious disease.^1^ Although much attention is given to its diagnosis and treatment, a growing body of evidence highlights the substantial proportion of human suffering that occurs after patients are considered ‘cured’. Recent data, quantified the long-term impact of TB in terms of disability-adjusted life years (DALYs), emphasizing the need to address post-TB lung disease (PTLD) as a critical public health issue.^2–4^ PTLD encompasses a spectrum of respiratory impairments that persist after microbiological cure for TB. These include chronic respiratory symptoms, airflow obstruction, restrictive lung disease, and bronchiectasis, leading to long-term disability and impaired quality of life.^5–8^ Patients continue to suffer from a wide range of respiratory (and non-respiratory) symptoms, and are often severely limited in their daily life and work activities.^5–10^ Despite its significant impact, PTLD has long been overlooked, with limited attention to systematic screening, diagnosis, and management at the programmatic level.

In recent years, the *International Journal of Tuberculosis and Lung Disease* (IJTLD) and *IJTLD Open* have published several of the milestone papers that have helped to shape our understanding of PTLD (see Table). This issue of *IJTLD Open* continues this trend with two key studies that further improve our understanding. A cohort study conducted in Kenya, Uganda, Zambia and Zimbabwe evaluated the feasibility of assessing and referring TB patients for comorbidities, risk factors, and disabilities within national TB programmes.^11^ Among 1,822 patients, 20% had significant disability (inability to walk 400 m in 6 min). Although referral rates for most conditions were high (85–100%), access to pulmonary rehabilitation services remained suboptimal. These findings underscore the need for integrated, patient-centred care models, particularly for rehabilitation. In addition, a follow-up study of 503 individuals who successfully completed TB treatment at 11 health facilities in China (2022–2023) sheds light on the long-term sequelae of TB.^12^ Alarmingly, 27% of patients remained disabled, 13% died or were lost to follow-up, and nearly half remained unemployed. These findings reinforce the urgent need to integrate long-term care strategies into TB programmes to address these ongoing health challenges. Both studies complement previous evidence, starting from the seminal work by Muñoz-Torrico et al.^13^ and symposia convened to discuss post-TB lung health,^14,15^ which established much-needed definitions and proposed diagnostic and management frameworks, laying the foundation for further research and clinical guidelines.^16–18^ The introduction of clinical standards provided a structured approach to identifying and managing PTLD, setting a precedent for national and international implementation.^16^ The Brazilian Thoracic Association (SBPT) and the Latin American Thoracic Association (ALAT) have since developed national and regional guidelines that bridge the gap between research and practice.^17–20^ WHO is currently working on a guidance document to provide a global framework for addressing PTLD in routine care settings, and recently published a policy brief on how to integrate the approach to TB and lung health.^8^

**Table.** Chronological order of milestone publications on PTLD.

Reference	Description
Muñoz-Torrico M, et al.^13^	First review of the available evidence on PTLD, including rehabilitation
Ravimohan S, et al.^9^	The prevalence of PTLD in non-MDR-TB patients varies widely, from 18% to 87%. This variation is due to differences in study populations and the pulmonary function tests used
Allwood B, et al.^14^	First International Symposium, with delegates from 13 countries across five continents, establishing a toolkit for future PTLD measurement
Migliori GB, et al.^16^	Definition of 6 clinical standards on patient evaluation, rehabilitation and patient education/counselling
Menzies NA, et al.^2^	In 2019, 58 million DALYs were attributed to post-tuberculosis sequelae globally. This is 47% of the total DALYs caused by TB disease
Maleche-Obimbo E, et al.^10^	Systematic review and meta-analysis showing that the prevalence of PTLD among patients with MDR-TB is high, ranging from 93.9% to 100%
Janete Inwentarz S, et al.^18^	Recommendations on PTLD from *Asociación Latinoamericana* de Tórax (ALAT)
Allwood BW, et al.^15^	Knowledge update on PTLD, identification of research priorities, and building research collaborations
Silva DR, et al.^21^	Largest study evaluating patients with PTLD who underwent pulmonary rehabilitation, and compared them with a cohort of non-rehabilitated patients

PTLD = post-TB lung disease; MDR-TB = multidrug-resistant TB; DALY = disability-adjusted life-years.

The clinical standards highlighted the challenge of the small sample sizes of many studies (typically fewer than 40 patients without control groups), the lack of clarity on how to integrate PTLD diagnosis and management into TB programmes, and the limited understanding of the role and practicality of pulmonary rehabilitation in PTLD care. However, recent research has started to fill these critical gaps. A major collaborative study in Brazil has provided robust evidence on the long-term burden of PTLD, answering many outstanding questions regarding feasibility and effectiveness, and showing the significantly better outcomes achieved by with rehabilitation.^21^ These findings, along with other insights,^22^ offer a clearer picture of post-TB care needs, although much more evidence is needed from the country experience.

Important projects are presently underway. Among them, as part of a TB REACH project, the Damien Foundation is implementing a programmatic approach to PTLD in Bangladesh, which includes rehabilitation.^23^ This aims at integrating the delivery of TB-associated disability and PTLD into the continuum of TB care. This includes prevention, screening, treatment support, and referral for pulmonary rehabilitation of TB-associated disability and PTLD. It will also include prevention and promotion activities, e.g., enhanced contact investigation for early TB diagnosis; TB prevention therapy (TPT), and smoking cessation.^23^ In Latin America ALAT, following its recent clinical guidelines,^18^ is developing a regional register of PTLD patients, allowing us to better understand both research and clinical needs. This growing body of evidence leaves no doubt: being declared ‘cured’ of TB does not mean the end of suffering for many people. The two studies presented in this issue of the Journal highlight the urgent need for systematic screening for PTLD (and other comorbidities) as part of TB treatment, strengthening referral pathways to ensure access to rehabilitation and chronic disease management, and embedding PTLD care into routine TB and primary healthcare services.

We intend for the *IJTLD* and *IJTLD Open* to continue to lead in this area and encourage you to send us your manuscripts and experiences of PTLD and its management. We also call on researchers, policymakers and clinicians to prioritize post-TB care and to ensure that the burden of PTLD is recognised at the global level. Only once this is achieved can we truly claim to be addressing the full impact of TB.
